# Compound Heterozygous Variants in the *IFT140* Gene Associated with Skeletal Ciliopathies

**DOI:** 10.3390/diagnostics14222601

**Published:** 2024-11-20

**Authors:** Katia Margiotti, Marco Fabiani, Antonella Cima, Antonella Viola, Francesca Monaco, Chiara Alì, Costanza Zangheri, Carmela Abramo, Claudio Coco, Alvaro Mesoraca, Claudio Giorlandino

**Affiliations:** 1 Laboratorio di Genetica Umana, Altamedica, Viale Liegi 45, 00198 Rome, Italy; marco.fabiani@artemisia.it (M.F.); antonella.cima@artemisia.it (A.C.); antonella.viola@artemisia.it (A.V.); francesca.monaco@artemisia.it (F.M.); chiaa.ali98@gmail.com (C.A.); costanza.zangheri@libero.it (C.Z.); claudio.coco@artemisia.it (C.C.); alvaro.mesoraca@artemisia.it (A.M.); caludio.giorlandino@artemisia.it (C.G.); 2 Ospedali Riuniti GOM Reggio Calabria, 89124 Reggio di Calabria, Italy; carmela.abramo@artemisia.it

**Keywords:** skeletal ciliopathies, clinical exome sequencing (CES), prenatal diagnosis

## Abstract

Ciliopathies are rare congenital disorders caused by defects in the structure or function of cilia, which can lead to a wide range of clinical manifestations. Among them, a subset known as skeletal ciliopathies exhibits significant phenotypic overlap and primarily affects skeletal development. This group includes several syndromes with overlapping but distinct clinical features, such as short-rib polydactyly syndrome (SRPS), Jeune asphyxiating thoracic dystrophy (JATD), Mainzer–Saldino syndrome (MZSDS), and cranioectodermal dysplasia (CED), also called Sensenbrenner syndrome. The most characterized features of skeletal ciliopathies are short stature, rhizomelic limb shortening, and thoracic narrowing to varying extents, with JATD presenting the most severe form. Here, we report a fetus with an extension of skeletal ciliopathy phenotype and compound heterozygous variants in the *IFT140* gene. The affected fetus had multiple malformations, including increased nuchal transparency (NT), shortened and thick long bones, hypoplastic tibia and fibula, absence of bladder, flat nose, and frontal bossing. Our findings expand the mutation spectrum of *IFT140*, and the clinical spectrum associated with skeletal ciliopathies, highly relevant in diagnosis prenatal settings.

**Figure 1:**
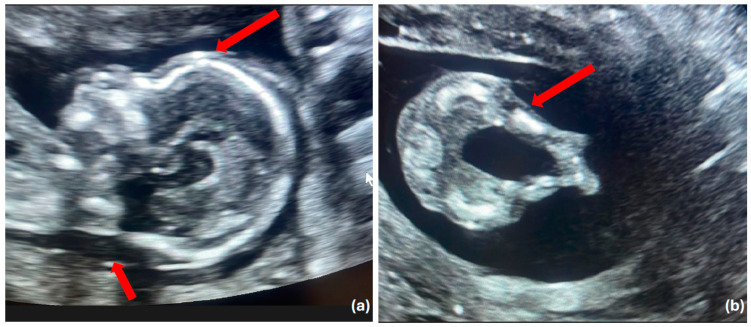
Prenatal ultrasound findings of fetus with skeletal ciliopathies. Skeletal ciliopathies represent a diverse group of disorders, including Jeune asphyxiating thoracic dystrophy (JATD), short-rib polydactyly syndrome (SRPS), Mainzer–Saldino syndrome (MZSD), and cranioectodermal dysplasia (CED). These conditions are typically characterized by a narrow chest with shortened ribs and polydactyly and often present with additional kidney, liver, and retinal diseases [1,2]. (**a**) Median sagittal ultrasound demonstrating increased nuchal translucency and frontal bossing. The increased NT, commonly associated with chromosomal abnormalities, when combined with the presence of frontal bossing, raises suspicion for more complex syndromic diagnoses, including ciliopathic disorders that affect both skeletal development and facial morphology (red arrows). (**b**) Transverse ultrasound showing short and curved femurs (red arrow). These findings are notable markers that may suggest an underlying skeletal ciliopathy [2]. A healthy 33-year-old multigravida woman was referred to our clinic at 17 weeks of gestation due to the presence of increased nuchal translucency measuring 5.1 mm. She had a history of three previous pregnancies, one of which ended in spontaneous abortion, another was terminated due to the presence of cystic hygroma, and one ended with a healthy child. She was otherwise healthy with no significant family history. Prenatal ultrasound revealed in addition to increased NT, shortened and thick long bones, hypoplastic tibia and fibula, referred absence of the bladder, a flat nose, and frontal bossing (Figure 1 and Figure 2).

**Figure 2:**
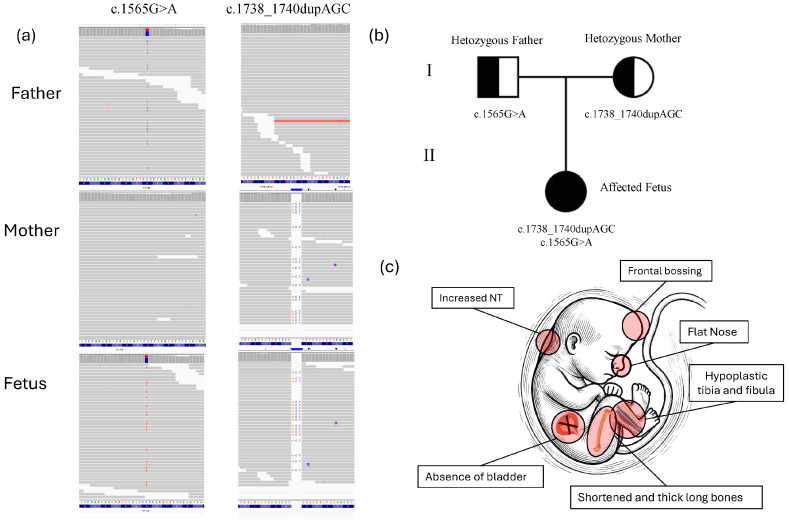
Prenatal ultrasound findings of fetus with skeletal ciliopathies. (**a**) Using fetal DNA obtained through amniocentesis, a normal karyotype and the absence of pathogenic copy number variants (CNVs) by array comparative genomic hybridization (aCGH) were assessed. Clinical exome sequencing (CES) and bioinformatic analysis revealed compound heterozygous mutations in the IFT140 gene. The heterozygous *IFT140* variant c.1738_1740dupAGC (p.Ser580dup) in one allele and a heterozygous IFT140 variant c.1565G>A (p.Gly522Glu) in the second allele, maternally and paternally inherited, respectively (Figure 2). Both variants were also confirmed by Sanger sequencing in fetus as well as in parents (Figure S1). The missense variant c.1565G>A (p.Gly522Glu) has previously been reported as a likely pathogenic variant in ClinVar (RCV001268554.8) in patients with Sensenbrenner syndrome, Jeune asphyxiating thoracic dystrophy syndrome, Mainzer–Saldino syndrome, and retinitis pigmentosa [3,4,5]. The duplication variant in *IFT140* c.1738_1740dupAGC (p. Ser580dup) is classified as a variant of uncertain significance (VUS) in ClinVar (VCV001426203.6) and has not been reported in other public databases, like HGMD Professional (accessed on 10 June 2024), or in individuals affected by IFT140-related conditions. This in-frame duplication results in the insertion of an additional serine amino acid at position 580 within the protein sequence. The insertion occurs in a region of the IFT140 protein that is important for its function, potentially altering the structural integrity and/or the functional dynamics of the protein. Furthermore, the detection of this variant in trans with a likely pathogenic variant, along with its compound heterozygous inheritance from two unaffected parents, provides evidence for its pathogenicity. According to the gnomAD database (as of 25 July 2024), the frequency of the p.Ser580dup variant is 0.000%, indicating it is extremely rare in the general population. This supports the application of ACMG criteria PM2 (extremely low frequency in population databases). Additionally, the compound heterozygous inheritance meets the criteria for PM3, and the observed duplication of a critical amino acid aligns with PM4, further strengthening the classification of the variant as likely pathogenic [6]. (**b**) Pedigree of the family. Half-filled symbols indicate carrier status for *IFT140* variants, roman numerals are used to indicate generations. (**c**) A minimalistic figure presents the fetus, emphasizing the clinical signs identified in the fetus. In particular, a prenatal ultrasound revealed, in addition to increased nuchal translucency (NT), markedly shortened and thickened long bones, hypoplastic tibia and fibula, suspected absence of the bladder, a flat nasal bridge, and pronounced frontal bossing. These skeletal abnormalities, along with facial dysmorphisms and potential visceral anomalies, strongly suggest a diagnosis consistent with a skeletal ciliopathy, a disorder typically characterized by defects in the structure and function of primary cilia affecting multiple organ systems [7]. The *IFT140* gene encodes the IFT140 protein, which is part of a large multi-protein complex known as the intraflagellar transport (IFT) complex. This complex plays a critical role in the formation, maintenance, and function of cilia—hair-like structures that extend from the surface of various cell types. The IFT140 protein is a component of the IFT-A complex, which primarily governs retrograde intraflagellar transport within cilia, facilitating the movement of cargo proteins from the tip of the cilium to its base. The *IFT140* gene comprises 31 exons (29 of which are coding) and encodes a protein of 1462 amino acids, featuring five WD repeats and nine tetratricopeptide (TPR) repeats [4,8,9]. Biallelic pathogenic variants in *IFT140* or other genes within the IFT-A complex can lead to defective retrograde ciliary transport. Such defects can result in a group of disorders called ciliopathies, which can impact multiple organ systems and cause a wide range of symptoms, including vision and hearing loss, skeletal abnormalities, kidney disease, and developmental delays [7,10]. More than 95 variants in the *IFT140* gene have been described in patients with Mainzer–Saldino syndrome or other short-rib thoracic dysplasia phenotypes, according to the HGMD database (2024.2 version). The rarest phenotype associated with IFT140-related ciliopathies is cranioectodermal dysplasia (CED). In the present study, the fetus was characterized by abnormalities of the long bones, hypoplastic tibia and fibula, flat nose, and frontal bossing with involvement of internal organs such as the refereed bladder absence. We characterized a novel heterozygous likely pathogenic variant p.Ser580dup in combination with a known likely pathogenic variant p.Gly522Glu in the *IFT140* gene. Additionally, we identified a new clinical feature associated with this phenotype, the absence of the bladder, which had never been previously reported in prenatal diagnosis of skeletal ciliopathies, whereas no other internal organ involvement was observed. The presence of shortened, thickened long bones, hypoplastic tibia and fibula, along with facial features such as frontal bossing and a flattened nose, aligns with the clinical features of short-rib thoracic dysplasia (SRTD), with or without polydactyly [11,12,13]. This group includes Jeune asphyxiating thoracic dystrophy (JATD), short-rib polydactyly syndrome (SRPS), Mainzer–Saldino syndrome (MZSD), and cranioectodermal dysplasia (CED), which exhibit diverse bone and/or cartilage abnormal phenotypes [14]. The identified genotype and ultrasonic skeletal features share several phenotypic features with previously reported patients carrying *IFT140* variants, particularly those associated with skeletal ciliopathies such as Mainzer–Saldino syndrome and Sensenbrenner syndrome [3,4,5] Similarly to these cases, our fetus exhibited shortened and thickened long bones, hypoplastic tibia and fibula, and facial dysmorphisms, including a flat nasal bridge and frontal bossing. The absence of the bladder observed in our fetus is a novel finding not previously reported in association with IFT140 mutations or skeletal ciliopathies in general. Previous studies have primarily reported renal anomalies such as cystic kidney disease or nephronophthisis in patients with *IFT140* variants [3,5,9]. In conclusion, genetic analysis, such as exome sequencing, is crucial in the prenatal diagnosis of skeletal ciliopathies. These conditions have overlapping clinical features, making differentiation challenging through ultrasound alone. Identifying genetic variants in genes like *IFT140* helps clarify the diagnosis, guide prognosis, and inform genetic counseling for affected families, providing critical insights for future pregnancy management. Reporting additional cases of fetal skeletal ciliopathies will aid in identifying genotype-phenotype correlations and pave the way for more accurate clinical diagnosis in prenatal settings.

## Supplementary Materials

The following supporting information can be downloaded at: https://www.mdpi.com/article/10.3390/diagnostics14222601/s1, Figure S1: Sanger sequencing of fetus confirming the two c.1738_1740dupAGC and c.1565G>A heterozygous variants in IFT140 previously identified by Next Generation Sequencing (NGS).
