# Supplementary material for: Compound Heterozygous Variants in the IFT140 Gene Associated with Skeletal Ciliopathies
Source: Diagnostics (Basel). 2024 Nov 20;14(22):2601. doi: 10.3390/diagnostics14222601 (PMC11593061; doi:10.3390/diagnostics14222601)
Supplement: Supplementary file 1 [file diagnostics-14-02601-s001.zip › diagnostics-3273849-supplementary.pdf]

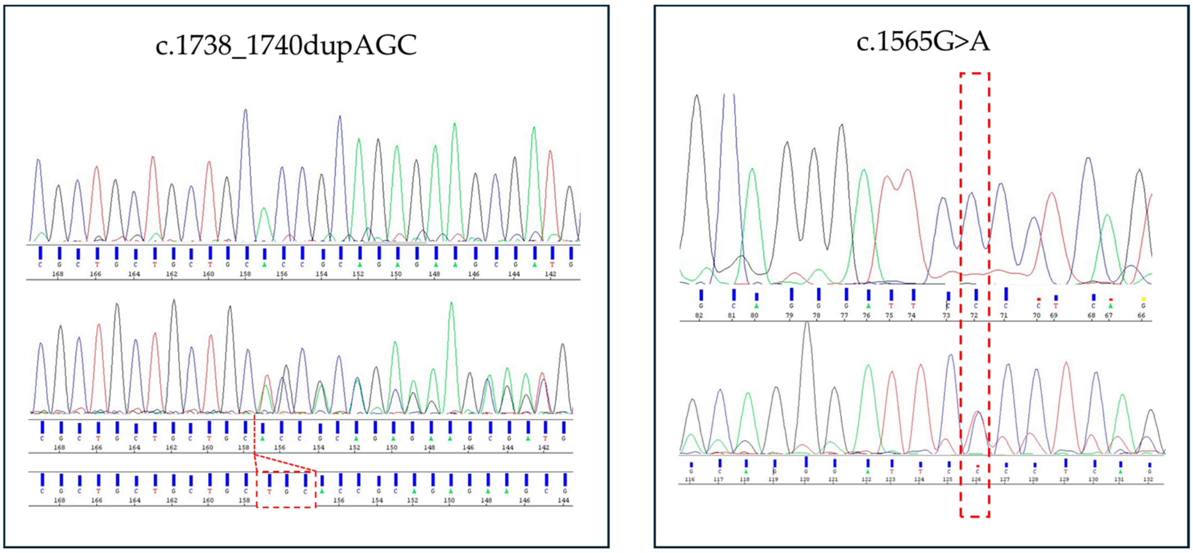

Figura S1: Sanger sequencing of fetus confirming the two c.1738_1740dupAGC and c.1565G>A heterozygous variants in IFT140 previously identified by Next Generation Sequencing (NGS)
